# How is an Electronic Screening and Brief Intervention Tool on Alcohol Use Received in a Student Population? A Qualitative and Quantitative Evaluation

**DOI:** 10.2196/jmir.1869

**Published:** 2012-04-23

**Authors:** Jessica Fraeyman, Paul Van Royen, Bart Vriesacker, Leen De Mey, Guido Van Hal

**Affiliations:** ^1^Epidemiology and Social Medicine, Medical Sociology and Health PolicyFaculty of Medicine and Health SciencesUniversity of AntwerpWilrijk (Antwerp)Belgium; ^2^Department of Primary and Interdisciplinary CareFaculty of Medicine and Health SciencesUniversity of AntwerpWilrijk (Antwerp)Belgium; ^3^Categorical Care CGGVagga vzw (Centre for Mental Health Care)AntwerpBelgium

**Keywords:** Alcohol, students, intervention

## Abstract

**Background:**

A previous study among Antwerp college and university students showed that more male (10.2%–11.1%) than female (1.8%–6.2%) students are at risk for problematic alcohol use. The current literature shows promising results in terms of feasibility and effectiveness for the use of brief electronic interventions to address this health problem in college and university students. We evaluated this type of intervention and cite existing literature on the topic.

**Objective:**

To develop a website, www.eentjeteveel.be, to motivate college and university students with problematic alcohol use to reduce alcohol consumption and increase their willingness to seek help.

**Method:**

The website contained a questionnaire (Alcohol Use Disorders Identification Test [AUDIT]) for students to test their alcohol use. According to their answers, the students immediately received personalized feedback (personal AUDIT score and additional information on risks associated with alcohol use) and a suggestion for further action. Afterward, students could send an email to a student counselor for questions, guidance, or advice. To obtain in-depth qualitative information on the opinions and experiences of students, we held 5 focus group discussions. The topics were publicity, experiences, impressions, and effects of the website. We analyzed the quantitative results of the online test in SPSS 15.0.

**Results:**

More than 3500 students visited www.eentjeteveel.be; over half were men (55.0%). A total of 34 students participated in the focus group discussions. The mixture of quantitative and qualitative methods to evaluate the intervention allowed a thorough analysis and provided complementary results. The intervention was well received by the student population. However, some minor aspects should be reconsidered, such as website publicity and providing students with options that were added after intermediate evaluation. The intervention increased the motivation of students to think about their alcohol use but could not stimulate them to change their behavior. The website attracted relatively more male than female students and more students in the high-risk group than in the low-risk group. The high-risk group was more inclined to seek advice or guidance (23/400, 6%; χ^2^_2 = 32.4,_ *P *< .001) than the low-risk group (34/1714, 2%; χ^2^_2 = 32.4,_ *P *< .001).

**Conclusions:**

We gained unique insight into students’ experiences, opinions, and perceptions with regard to the intervention. The results show that the intervention was positively received in the population, and the willingness to seek help was increased. However, real behavior change needs further research. The results of this study can assist health providers and researchers in better understanding college and university students’ perceptions of eHealth initiatives.

## Introduction

The Internet is an ever-present and increasingly important aspect of modern society. Therefore, online interventions are becoming a more important way of approaching young people on health issues. Young people see the Internet as an acceptable and credible source of health information. College and university students are often reluctant to participate in face-to-face motivational counseling on their alcohol use. Students at high risk for problematic alcohol use are rarely interested in attending individual treatment or group programs [1] and prefer self-help interventions without therapeutic involvement to address their problem drinking [2]. Previous research has shown that students tend to prefer electronic counseling and feedback over face-to-face contact with professional counselors concerning their alcohol use [3].

A recent systematic review of the drinking patterns of European university students concluded that alcohol is consumed by a large group of students, which increases the risk of problematic drinking, making it an important issue for prevention [4]. More specifically, heavy drinkers, who are frequently targeted in prevention campaigns are also often not eager to seek help because they don’t consider their drinking behavior problematic and, moreover, drinking is even seen as normal student behavior [4-6]. When heavy drinkers do seek help, they prefer low-threshold interventions such as computer-based interventions [7]. An electronic screening and brief intervention tool can offer several options for constructing such a computer-based intervention with a low threshold. In a search of the current literature, we found a variety of examples of interventions using email [8,9], a computer in a community health center [10], or a website [9,11-16] as key elements. Compared with the other options, Internet interventions present few barriers and keep the threshold for participation low.

Additionally, research indicates that male students are more at risk than women for harmful alcohol use (drinking larger quantities and on more occasions) [4], and women are more willing than men to seek help [17]. Therefore, developing an intervention that takes into account this gender issue presents an additional challenge.

Aside from lowering the participation threshold for interventions, electronic interventions offer several other advantages. They are perceived as more anonymous than face-to-face contact and create a safer environment for the user, thus minimizing the bias of giving socially acceptable answers [12]. Because of this advantage, electronic interventions are suitable for target populations with limited insight into their own problematic behavior and for people who do not seek help due to stigmatization [18,19]. Furthermore, online interventions are suitable for people who are not highly motivated to live a healthy lifestyle [19]. Also, previous research has shown that an electronic screening and brief intervention tool in particular can effectively increase the motivation of high-risk student drinkers to change their drinking behavior [9]. In general, online interventions that target alcohol consumption can be especially beneficial for at-risk drinkers and young people [20]. Current technologies make it possible to extend the intervention with movies, games, or simulations. In this way, an electronic intervention can become more attractive to young people. Moreover, the Internet makes it possible to reach a large sample of people in a relatively easy way and in a short period of time. Perhaps the biggest disadvantage of this approach (universal prevention) is the lack of personal contact, making verbal communication impossible (on such a large scale) [21].

The literature shows that a brief electronic intervention is significantly more effective in altering drinking behavior than no intervention at all [12,21]. Most of the existing initiatives consist of websites that provide a screening instrument and personalized feedback. The effectiveness of this type of intervention is independent of the feedback delivery mode (email or personal contact) [12,15]. The content or type of feedback differs: blood alcohol concentration, risk factors for alcohol-related harm, negative consequences of alcohol use, impact on surroundings, impact on sex life, or pointers for becoming a moderate drinker [15]. Another option is to offer personalized feedback on drinking norms, which appears to be effective and feasible [9] but needs further investigation [12,22].

Electronic interventions are positively experienced overall within a student population [9,12]. Various randomized controlled trials show that electronic interventions aimed at students in higher education can decrease the negative effects of alcohol use within this population [1,10,12-14,23]. Several other studies have shown a decrease in weekly alcohol consumption [24-26], heavy drinking [25,27], or average alcohol consumption [28] after implementation of an electronic intervention. This variety of outcome measures in research evaluating the effect of electronic screening and brief intervention tools does not, however, allow general statements to be made [20]. Although these results on the effectiveness of these tools are positive, more research is needed. Several literature reviews address the need for more methodological rigor to underpin statements about the effectiveness of electronic screening and brief intervention tools [29], online interventions [2,20], or computer-based interventions in general [21,25].

Next to differences in outcome measurements, another methodological issue is the lack of long-term follow-up measurements [20]. Moreira et al [22] reached a similar conclusion in a Cochrane review of the use of the social norms approach to reducing alcohol use in a student population. In a qualitative review, Elliot et al [21] advocated more studies over a longer stretch of time, assessing the influence of psychological factors on the effectiveness of e-interventions. The general results of these reviews were, however, in favor of electronically delivered interventions.

We designed an electronic screening and brief intervention to screen students with hazardous or problematic alcohol use. The aim of the intervention was to motivate students to reduce their alcohol consumption and to increase their willingness to seek help. In this study, we assessed the following research questions. How is an electronic intervention received in a student population? Which group of students is reached by the intervention? What are students’ experiences with the intervention? How can the intervention motivate students at risk for problematic alcohol use to think about their alcohol use and to change their drinking behavior?

## Methods

### Development of the Intervention

We developed a website (www.eentjeteveel.be in Dutch, “one too many” in English) to spread the intervention throughout the student population in Antwerp (the university and all institutions for higher education). The website comprised two major parts: an information section and the screening tool. The information section informed the visitors of the prevalence of alcohol use among students, the risks of alcohol use, contact details for further help or advice, the background of the screening tool, and the organizing research team.

Students were also provided with some pointers to help friends who are at risk for hazardous or problematic alcohol use. This part briefly pointed out different ways to recognize possible problematic drinking behaviour and how to respond. Frequently asked questions and existing misperceptions surrounding sexuality and alcohol use were also discussed. To determine the reach of this intervention, we asked visitors to respond to the following questions. “Is this the first time you have visited the website? (Yes/No).” In this way, we could record repeat visits. “Are you a student in Antwerp? (Yes/No).” We also asked visitors to fill in their age and gender. The screening tool assessed drinking behavior and included the 10 questions of the Alcohol Use Disorders Identification Test (AUDIT) [30]. This validated screening instrument has been identified as a useful and reliable tool for detecting alcohol abuse and dependence among college students [31]. Subsequently, we asked students to fill in the number of standard drinks (defined as 10 g ethanol/drink, as in European standards [32]) they consumed per day in a normal week. According to the sensible drinking guidelines from the British Medical Association (BMA), harmful drinking can be assessed in terms of the fact that more than 21 standard drinks per week for males (210 g ethanol or 26.5 units in the United Kingdom) and 14 standard drinks for females (140 g ethanol or 17.5 units) can cause physical damage due to alcohol intake. In total, the tool consisted of 16 questions as described above (including gender, age, and student campus).

Immediately after finishing the test, respondents were shown their personalized feedback. This feedback consisted of the respondent’s personal AUDIT score and a standard AUDIT score table [33]. According to this personal score, additional information was added to inform the respondents about the risks of their alcohol intake. Respondents who scored 8 or more on the AUDIT or who exceeded the weekly standard guidelines as set out by the BMA were shown a table with health-related risks due to alcohol intake.

Specifically for the student visitors, we offered 5 options for further follow-up, based on the theoretical model of change [34]. Students who were not ready for follow-up or dropped out from the change model could stop the test and were referred back to the homepage. Students who wanted more information on alcohol use and the related risks were referred to the information section of the website. If students were ready to take further action, they could choose among the following options. “Where can I get some more advice?” “Can I talk to a student counselor?” “I’m ready to stop drinking/reduce my drinking; can I get some guidance from a student counselor?”

Students who wanted to take further action were referred to another webpage where a fill-in form was shown. This form gave them the chance to ask a more specific question directed at a student counselor of their choice. Student counselors, mostly psychologists, provide specific counseling and guidance on mental health and specific study problems. A list was shown of all student counselors on all college and university campuses (a picture of every student counselor and all contact details were shown). Additionally, an option was added to send the feedback to their personal email address, to the student counselor of their choice, or both. The first option gave the students the opportunity to take further action later. A copy of the feedback was sent to their personal mailbox with a link back to the website. Personal data (email addresses and names) were treated with discretion and were not stored on the Web server, due to privacy concerns expressed by the participating university and colleges for higher education. The questions posed by the students who chose to contact a student counselor, the name of the selected student counselor, and the personalized feedback were recorded in the data file. No consent was required for entrance to the website (which was freely available).

We informed students about the website and the intervention through a range of different channels: posters and flyers, messages on the digital platforms (websites that require a login used for offering students information on the courses of their study subject, eg, slides, and exams or other student activities), websites of student clubs and student counseling services, messages in student magazines, person-to-person, and direct referrals from counselors to the website. In addition to these ongoing promotion initiatives, two reminders were sent at the beginning and in the middle of the academic year, by sending messages to all student email addresses provided by the colleges and university for every student.

The intervention was evaluated after being online for 1 year (April 2008 to April 2009). We made important adjustments after an intermediate evaluation in October 2008. A list with contact details (including pictures) of all student counselors was added and the option was given to forward the personalized feedback to an email address of choice. These adjustments aimed to lower the threshold for students to contact a student counselor for help or advice.

### Evaluation Method

The intervention was evaluated in two different ways intended to be complementary. First, we analyzed the data that were recorded in the online screening tool as students filled in the questionnaire. These quantitative data were obtained in Excel (Microsoft Corporation, Redmond, WA, USA) and descriptive analyses (frequencies and cross-tabulations) were performed in SPSS 15.0 (IBM Corporation, Somers, NY, USA).

Second, we explored in depth the experiences, opinions, and perspectives of college and university students on the appearance, content, and effect of the intervention, which had not previously been explored.

### Focus Group Discussion Method

Focus group discussions are a highly appreciated and valuable method for exploring the complex composition of experiences, opinions, and perspectives [35-37]. Research has shown that people are more inclined to discuss their experiences in a group than in a face-to-face approach [38]. All focus group discussions were moderated by an experienced moderator with thorough knowledge of motivational interviewing. The ability of the moderator to stimulate discussion and to guide the conversation in the right direction is essential in focus group discussions [38].

A semistructured interview guide of open questions was used to carefully elicit the experiences, opinions, and perspectives of the participants and to stimulate discussion. The interview guide consisted of an introduction (where confidentiality issues were explained), transitional questions, key questions, and subquestions (see Multimedia Appendix 1). The key question of the interview guide for the focus group discussions was “How did the intervention help you think about your alcohol use and change it?” This question was not used in the focus group discussions as such (see further details in Multimedia Appendix 1). Most themes were set out beforehand in the interview guide and concerned the general evaluation aspects of the interventions: knowledge of and participation in the website, positive and negative experiences with the website, and general results of the intervention relating to alcohol use among college and university students. Some other themes that emerged during the first focus group discussions (as suggested by the participants) were added to the script and used in the following focus group discussions (see Multimedia Appendix 1).

The composition of the discussion groups needs to meet the criterion of homogeneity. During the discussion, participants need to feel that they are among equals to be able to speak freely about their personal experiences. We randomly invited college and university students from Antwerp who were in the first 3 years of their educational program based on their willingness to participate in the discussion group (by email or personal invitation). Previous studies have shown that this younger group of students is more at risk for problematic alcohol use [39,40]. Convenience sampling was applied [37]. Another criterion for inclusion was participation in the intervention (visited the website and took the test), and we strived for an equal distribution by gender and study subject in the focus group discussions. We set a minimum of 4 and maximum of 10 participants for the focus group discussions. The number of focus group discussions is determined by saturation of the data (no new results can be obtained by adding a focus group discussion), which is usually reached after 4 or 5 focus group discussions. Representativeness of the participants is not a criterion in focus group discussions [38].

All the focus group discussions were audiorecorded digitally and transcribed verbatim. JF and BV independently coded all transcripts. Through an iterative process of constant comparison and reflection, we obtained a frame of codes giving insight into and explaining the perception of students regarding the electronic screening and brief intervention tool and the questions in the script. The applied method was inductive content analysis, which is commonly used in qualitative research [37]. In this paper we present a selection of the most important themes (a complete report in Dutch can be obtained from the corresponding author). Results of the analysis are illustrated with relevant quotes from the students in the focus group discussions.

## Results

### Quantitative Evaluation

Between April 7, 2008 and April 6, 2009, a total of 5664 people visited the website, 62.29% (n = 3528) of them students in Antwerp. More than half of the students who visited the website were male (n = 1936, 54.88%). The majority visited the website only once (n = 3395, 96.23%). The mean age was 21 (SD 3.8) years with a minimum age of 15 years and a maximum of 58 years. The mean age of all Flemish (from Flanders, the northern Dutch-speaking part of Belgium; Antwerp is situated in the center of Flanders) college and university students in the academic year 2007–2008 was 21 years, with a minimum age of 16 years and a maximum of 73 years [40]. In this study we obtained a representative sample of college and university students with regard to age.

On average, 10.39% of the students per participating institution for higher education visited the website at least once (3395 student visitors/33,222 students in the participating institutions). The proportion of males to females (54.55% and 45.45%, respectively) in the sample differed from the proportion in the population of Antwerp students (45.83% and 54.17%, respectively) (see Table 1). Thus, we reached proportionally more male than female students.

**Table 1:** Gender division for total number of college and university students in Antwerp and the students who visited the website www.eentjeteveel.be.

Gender	Number of visitors to the website		Total number of college and university students in Antwerp	
	n	%	n	%
Male	1852	54.55%	15,226	45.83%
Female	1543	45.45%	17,996	54.17%
Total	3395	100.00%	33,222	100.00%
Missing^a^	133	3.8%	0	0%

^a ^These students did not fill in the question on gender (missing data not in total count).

Of the 3213 students who participated in the intervention, 41.15% (n = 1322) were at low risk for problematic alcohol use (score of 0–7 on the AUDIT), 40.34% (n = 1296) were at medium risk (score of 8–15 on the AUDIT), 9.7% (n = 311) were at high risk (score of 16–19 on the AUDIT), and 8.8% (n = 284) were at very high risk (AUDIT score of 20 or more) [30]. More male (481/1767, 27.3%) than female students (114/1446, 8.0%) were at high to very high risk (see Table 2). A relationship is observed between AUDIT scores and the results from the BMA guidelines. The results show that, when the threshold amount for hazardous drinking is exceeded, students have a high risk of problematic alcohol use (score of 16–40 on the AUDIT).

**Table 2:** Alcohol Use Disorders Identification Test (AUDIT) score versus gender.

AUDIT score range	Male students		Female students		All students	
	n	%	n	%	n	%
0–7^a^	446	25.2%	876	60.6%	1322	41.15%
8–15^a^	840	47.5%	456	31.5%	1296	40.34%
16–19^a^	254	14.4%	57	4%	311	9.7%
20–40^a^	227	12.9%	57	4%	284	8.8%
Missing^b^	0	0%	0	0%	297	9.2%
Total	1767	100.00%	1446	100.00%	3213	100.00%

^a ^Statistically significant for χ^2^_3 = 452.6,_ *P *< .0001.

^b ^These students did not complete the test (missing data not in total count).

Of the students who did the test more than once, a larger proportion reported a higher risk for problematic alcohol use (score of 16–40 on the AUDIT) (n = 36, 29%; χ^2^_1 = 9.5,_ *P *< .0001) than during their first visit (n = 559, 18.1%; χ^2^_1 = 9.5,_ *P *< .0001) (see Table 3).

**Table 3:** Alcohol Use Disorders Identification Test (AUDIT) score for college and university students during their first or repeat visit to the website www.eentjeteveel.be.

AUDIT score range	First visit^a^		Repeat visit^b^		Total visits	
	n	%	n	%	n	%
0–15^c^	2530	81.90%	88	71%	2618	74.21%
16–40^c^	559	18.1%	36	29%	595	16.9%
Missing^d^	0	0%	0	0%	315	8.9%
Total	3089	100.00%	124	100.0%	3528	100.00%

^a ^Counts all records with answer of yes to the question “Is this the first time you have visited the website? (Yes/No).”

^b ^Counts all records with answer of no to the question “Is this the first time you have visited the website? (Yes/No).”

^c ^Statistically significant for χ^2^_1 = 9.5,_ *P *< .002.

^g ^These students did not complete the test.

Of 2114 students who completed the test and chose 1 of the follow-up options, 82.73% (n = 1749) chose to return to the homepage and quit the intervention. Almost 15% of the students (n = 308, 14.6%) preferred to look for more information on their alcohol use and 3% (n = 57) chose a follow-up action (advice, appointment, or guidance) (see Table 4). In total, 3 students sent an email to 1 of the student counselors. Of these, 2 wanted to receive guidance in reducing drinking and 1 student asked to make an appointment with a professional. The researcher consulted the student counselors who were contacted by the students to verify whether a follow-up took place after the student counselor received an email from the student through the website (the student’s privacy was ensured during this process). All 3 students received a request for an appointment from the student counselor, as described by the standard procedures. In none of the cases was further follow-up achieved.

**Table 4:** Alcohol Use Disorders Identification Test (AUDIT) score for college and university students vs. choices for follow-up.

Choice	AUDIT score range				All students	
	0–15		16–40			
	n	%	n	%	n	%
Stop the test^a^	1452	84.71%	297	74.3%	1749	82.73%
More information^a^	228	13.3%	80	20%	308	14.6%
Further action^a^	34	2%	23	6%	57	3%
Missing (no action)^b^	0	0%	0	0%	1117	
Total	1714	100.0%	400	100.0%	2114	100.00%

^a ^Statistically significant for χ^2^_2 = 32.4,_ *P *< .001.

^b ^These students did not choose 1 of the follow-up options, but did finish the test (not in total count).

There was an association between AUDIT scores and the choices for follow-up. Most of the students (1452/1714, 84.71%) in the low-risk group (score of 0–15 for the AUDIT) chose to stop the test. Proportionally fewer students (297/400, 74.3%) from the high-risk group (score of 16–40 for the AUDIT) also chose to stop the test. More students from the high-risk group (n = 80, 20.0%) than the low-risk group (n = 228, 13.3%) chose to get more information on their alcohol use. The outcome was similar for the follow-up actions: 6% (n = 23) of the high-risk group and 2% (n = 34) from the low-risk group chose a follow-up action (advice, appointment, or guidance) (see Table 4).

### Qualitative Evaluation

In February and March 2009 (during the final phase of the online intervention), we carried out 5 focus group discussions with 17 male and 17 female students between 18 and 23 years old, with a mean age of 19. All participants were enrolled as bachelor’s students at the Antwerp University or at one of the six participating institutions for higher education in the city of Antwerp. They were invited to visit the website before participating in 1 of the focus group discussions. Students in the focus group discussions indicated a mean alcohol intake of 7 standard drinks per week. A total of 4 students reported no drinking, 4 female students reported an average of 7 to 14 standard drinks per week, and no female students reported an average of more than 14 alcoholic beverages per week. Among the male students, 8 drank on average between 7 and 21 standard drinks per week, and only 1 reported an average of more than 21 alcoholic beverages per week (see Table 5).

**Table 5:** Characteristics of participants in the focus group discussions.

Focus group	Gender	Age (years)	Standard drinks/week	Study subject
1	Male	20	8	Nautical sciences
	Male	18	2	Nautical sciences
	Female	18	1	Nautical sciences
	Male	18	0	Nautical sciences
	Male	19	15	Nautical sciences
	Male	20	6	Nautical sciences
	Male	19	20	Nautical sciences
	Male	19	3	Nautical sciences
2	Male	21	8	Social studies
	Female	23	6–10	Social studies
	Female	19	7–14	Social studies
	Male	19	17	Social studies
	Female	22	0	Social studies
	Female	19	6	Social studies
	Female	23	5–8	Social studies
	Female	22	6–10	Social studies
3	Male	21	0	Social studies
	Male	21	30	Social studies
	Male	22	20	Social studies
	Female	19	5	Social studies
	Male	20	8	Social studies
	Male	22	7	Social studies
	Female	21	7	Social studies
4	Female	20	14	Veterinary medicine
	Female	19	6–7	Veterinary medicine
	Female	19	1–2	Veterinary medicine
	Female	20	2–3	Veterinary medicine
5	Female	18	2–3	Applied psychology
	Male	18	0	Applied psychology
	Female	18	4	Applied psychology
	Male	20	4	Applied psychology
	Female	18	3	Applied psychology
	Female	18	6	Applied psychology
	Male	19	3	Applied psychology

#### General Experiences With the Website

Generally, students in the focus group discussions experienced their visit to the website as positive. According to the students, the website offered clear information about their alcohol use, as illustrated in the following quotes (Q).

Q1: The name of the website is well chosen, it sounds nonjudgmental.

Q2: I found the pointers for friends very useful. This way, the website aims not only at people with an alcohol problem, but also at people surrounding them. That can be useful.

Q3: I’ve found some references for further help. That’s a positive thing.

#### Evaluation of Alcohol Use and Related Risks

This theme emerged spontaneously during the focus group discussions when exploring the experiences of the students with the website. Most students found it difficult to evaluate their own alcohol intake and related risks (Q4). Some felt that they had a realistic idea of their own drinking habits (Q5). However, they generally underestimated their drinking behavior (Q6, Q7). Indeed, when receiving the personal feedback, some students did not believe the results that were shown (Q7).

Q4: You think to yourself “I don’t drink too much” and “it causes no harm,” but then it appears that it does...

Q5: I think everyone can evaluate his own drinking behavior; at least I can.

Q6: I found the results of the test interesting because I had a score of 7. A score of 8 would have put me in the second risk category. That made me think for a second: “maybe I should be aware...” Because I expected a lower score.

Q7: When I saw my results, I thought “Oh, I did something wrong, this can’t be right.”

Students also underestimated the general risks of excessive alcohol use (Q8, Q9).

Q8: Maybe I drink 35 units on Friday and Saturday, and zero units the rest of the week. That’s not addictive behavior in my opinion, or is it?

Q9: The possible consequences seem so far away, you can’t see them...

#### Perceived Goal and Target Group of the Website

In general, students did not feel that the intervention was addressing them. The website can be useful only for people who have doubts about the amount of their own alcohol intake and people who are already planning to seek help or advice (Q10). The website cannot help people who are not aware of any existing alcohol-related problems, according to the students (Q11).

Q10: For someone who has the feeling “maybe it’s getting to be a bit too much,” the website can have an impact maybe.

Q11: The people with the biggest problem—the ones who don’t care but are not aware of the problem—are the most difficult ones to get on the website.

#### Usefulness of the Website for Others

Mostly, the students reflected on possible negative consequences of alcohol use for others rather than for themselves. They spontaneously mentioned the importance of their own role as a friend in dealing with problematic alcohol use in their friends (Q12, Q13). Additionally, the website could provide useful information for friends, parents, and youth workers (Q14).

Q12: You have to be honest with your friends about their alcohol use.

Q13: If one of your friends comes up to you and says ”I have a drinking problem”...yeah, I would refer her, I think.

Q14: ...you can fill in the test with leaders from youth movements, people who work with young people, so they can sensitize young people in their own context.

#### Motivation to Change

Most of the students said that the intervention did not motivate them to change their alcohol use, as a second step in behavioral change (Q15). However, when their test results were more alarming, they would be stimulated to think about it, as a first step in behavioral change (Q16).

Q15: Now, when I start my fourth drink, I think “Ow, in fact, maybe four drinks is a bit too much,” but I don’t think [my drinking behavior] can change because of the website...

Q16: I had a score of 8 on the test and I drink a glass of wine every night with dinner. If my score had been higher, I would consider drinking less. Just realizing that all sorts of bad things can happen to your liver...

The students in the focus group discussions generally considered their own alcohol use not to be problematic, although at least one student did show risk for harmful alcohol use according to the BMA guidelines (see Table 5). They were stimulated to think about the alcohol use of their friends more than about their own alcohol use (Q17). A few students would consider talking about their alcohol use after visiting the website (Q18).

Q17: [The score] worried me, especially for my friends. Because I’m not a heavy drinker myself, and when I saw my score, I thought “Wow, my friends, if they did this test, they would score even higher!”

Q18: You could talk with your parents about it, using the information from the website.

## Discussion

An electronic screening and brief intervention was developed, aimed at Antwerp college and university students, to test their alcohol use and to gain insight into their drinking behavior by offering an easily accessible tool. This is the only existing electronic screening and brief intervention tool in Flanders and Belgium specifically focusing on college and university students.

Although on average 10.39% of the students per participating institution for higher education in Antwerp visited the website, publicity for the website is a rather weak point. The sample of students that we obtained in the online intervention was representative according to age; however, we did not consider other dimensions for representativeness. During the focus group discussions, students stressed the lack of publicity initiatives for the website. We set up two major campaigns to reach students during the college year. These initiatives had a positive influence. However, we saw a steep decline in visitor numbers between and shortly after these initiatives. More persistent and repeated actions are needed to maintain students’ attention, for example, advertising on social network sites such as Facebook and LinkedIn.

Only 3 students took further action and sent an email to a student counselor. We cannot know how many students approached the listed student counselors by personal or telephone contact following a visit to the website. After visiting the website, students could have approached other health services outside the possibilities offered in this intervention; however, it was not our intention to assess this.

Additionally, possibly relevant data were missed by not recording the number of times that students had personal feedback sent to their own email address. These results could inform us about the students and their intention to change their drinking behavior.

Although this study has some limitations, our results can offer social scientists and health workers insight into the experiences and attitudes of students toward an electronic screening and brief intervention aimed at reducing alcohol drinking among those with problematic or hazardous drinking behavior. Until now, this was an unexplored topic.

The results of the study show that students in high-risk groups are more inclined than those in low-risk groups to seek help or advice. This is confirmed in the qualitative research, where students indicated that they would be more motivated to take further action when the feedback showed alarming results. Also, more students from the high-risk group repeatedly visited the website. We can therefore conclude that students were motivated to think about their alcohol use after participating in the intervention and that this motivation was even increased when the risk for problematic alcohol use increased. These findings are in accordance with several other studies [9,12,20]. However, more research on the nature of this relationship is needed.

A previous study among Antwerp students showed that more male (10.2%–11.1%) than female (1.8%–6.2%) students are at risk for problematic alcohol use [16]. A recent study among Antwerp and Ghent students showed the same results (11.1% male and 1.7% female problematic alcohol users) [40]. A review on drinking in European universities found similar results [4]. Moreover, women are more willing than men to seek help [17]. In the quantitative analysis we found that more male than female students visited the website. Also, the proportion of students in high-risk groups was higher than in the previous study by Van Hal et al [39]. Therefore, the intervention succeeded in meeting the challenge to reach more men than women and more students at high risk than at low risk for problematic alcohol use. However, the selection of the cut-off points in the AUDIT score and the BMA standard to determine different risk groups is influenced by national and cultural standards, which are also determined by maximum consumption allowances [30]. In the United Kingdom, the cut-off point for the number of weekly standard drinks is 110.6 g (7.9 g × 14 drinks) of ethanol per week for women and 165.9 g (7.9 g × 21 drinks) or ethanol for men, compared with 140 g and 210 g, respectively, in Belgium. The results in this study, therefore, may imply an underestimation compared with harmful drinking in the United Kingdom.

With regard to the average alcohol intake per week reported by the participating students, we unintentionally gathered a mixed group of drinkers in every discussion group. Although it was not the intention of the study to gather a representative group of students with regard to alcohol intake, this can be considered a strength of the study. Heavy drinkers as well as abstainers participated in the focus group discussions and shared their opinions in the discussion. However, this can also be seen as a weakness of the study. The intervention aimed to address especially high-risk drinkers, and the group of participants in the focus group discussions was mixed with regard to alcohol use.

The role of friends in the management of alcohol use is important. Students were more likely to overestimate the alcohol intake of other students and to underestimate their own alcohol use, creating a situation where students are at risk for excessive alcohol intake. Previous research has indicated that perceived social norms are a realistic predictor of future behavior in students [41-44] regardless of gender [45]. Misperceptions can be tackled by correcting the perceived social norms [23,43,44,46-48]. Adding extra information to the personalized feedback in the intervention according to the social norms approach can address these misperceptions in the future.

The combined use of both quantitative and qualitative methods makes the current study highly valuable and rare within this subject area. On the one hand, the quantitative evaluation gives a good overview of the participation of the student population in the intervention and can show us trends in the status of alcohol use among the student population who participated in the intervention. On the other hand, the exploratory method of focus group discussions gives us a unique insight into the experiences, opinions, and perceptions of the same population on the intervention, which cannot be obtained in any other scientifically justified manner.

### Conclusion

Recent studies have shown the effectiveness of freely available and anonymous online interventions in reducing alcohol use in student populations. To demonstrate the effectiveness of our intervention, a follow-up study needs to be performed with one or more control groups and one intervention group. However, this study shows that qualitative methods can be used in a pragmatic and a scientifically rigorous manner simultaneously. The results show that the intervention was well received in the student population, although most students did not feel addressed by it. It reached more male than female students and also more students in high-risk groups. The results show that the willingness to seek help increases when the risk for problematic alcohol use increases. However, the impact of the website on real behavior change needs further research. We were able to gain highly valuable information that deepens the knowledge base on the feasibility of an eHealth brief intervention tool for alcohol use aimed at college and university students. The results of this study can assist health providers and researchers in better understanding the perceptions of college and university students with regard to eHealth interventions.

## Multimedia Appendix 1

Script for focus group discussions.

## Abbreviations

AUDIT: Alcohol Use Disorders Identification Test
BMA: British Medical Association

## References

[1] Chiauzzi E, Green TC, Lord S, Thum C, Goldstein M (2005). My student body: a high-risk drinking prevention web site for college students. J Am Coll Health.

[2] Riper H, van Straten A, Keuken M, Smit F, Schippers G, Cuijpers P (2009). Curbing problem drinking with personalized-feedback interventions: a meta-analysis. Am J Prev Med.

[3] Kypri K, Saunders JB, Gallagher SJ (2003). Acceptability of various brief intervention approaches for hazardous drinking among university students. Alcohol Alcohol.

[4] Wicki M, Kuntsche E, Gmel G (2010). Drinking at European universities? A review of students' alcohol use. Addict Behav.

[5] Cho H (2006). Readiness to change, norms, and self-efficacy among heavy-drinking college students. J Stud Alcohol.

[6] Cellucci T, Krogh J, Vik P (2006). Help seeking for alcohol problems in a college population. J Gen Psychol.

[7] Buscemi J, Murphy JG, Martens MP, McDevitt-Murphy ME, Dennhardt AA, Skidmore JR (2010). Help-seeking for alcohol-related problems in college students: correlates and preferred resources. Psychol Addict Behav.

[8] Moore MJ, Soderquist J, Werch C (2005). Feasibility and efficacy of a binge drinking prevention intervention for college students delivered via the Internet versus postal mail. J Am Coll Health.

[9] Bendtsen P, Johansson K, Akerlind I (2006). Feasibility of an email-based electronic screening and brief intervention (e-SBI) to college students in Sweden. Addict Behav.

[10] Kypri K, Saunders JB, Williams SM, McGee RO, Langley JD, Cashell-Smith ML, Gallagher SJ (2004). Web-based screening and brief intervention for hazardous drinking: a double-blind randomized controlled trial. Addiction.

[11] Cunningham JA, Humphreys K, Koski-Jännes A, Cordingley J (2005). Internet and paper self-help materials for problem drinking: is there an additive effect?. Addict Behav.

[12] Bewick BM, Trusler K, Mulhern B, Barkham M, Hill AJ (2008). The feasibility and effectiveness of a web-based personalised feedback and social norms alcohol intervention in UK university students: a randomised control trial. Addict Behav.

[13] Kypri K, Langley JD, Saunders JB, Cashell-Smith ML, Herbison P (2008). Randomized controlled trial of web-based alcohol screening and brief intervention in primary care. Arch Intern Med.

[14] Walters ST, Vader AM, Harris TR (2007). A controlled trial of web-based feedback for heavy drinking college students. Prev Sci.

[15] Walters ST, Miller E, Chiauzzi E (2005). Wired for wellness: e-interventions for addressing college drinking. J Subst Abuse Treat.

[16] Kypri K, Stephenson S, Langley J, Cashell-Smith M, Saunders J, Russell D (2005). Computerised screening for hazardous drinking in primary care. N Z Med J.

[17] Benton SL, Benton SA, Downey RG (2006). College student drinking, attitudes toward risks, and drinking consequences. J Stud Alcohol.

[18] Copeland J, Martin G (2004). Web-based interventions for substance use disorders: a qualitative review. J Subst Abuse Treat.

[19] Van 't Riet J, Crutzen R, De Vries H (2010). Investigating predictors of visiting, using, and revisiting an online health-communication program: a longitudinal study. J Med Internet Res.

[20] White A, Kavanagh D, Stallman H, Klein B, Kay-Lambkin F, Proudfoot J, Drennan J, Connor J, Baker A, Hines E, Young R (2010). Online alcohol interventions: a systematic review. J Med Internet Res.

[21] Elliott JC, Carey KB, Bolles JR (2008). Computer-based interventions for college drinking: a qualitative review. Addict Behav.

[22] Moreira MT, Smith LA, Foxcroft D (2009). Social norms interventions to reduce alcohol misuse in university or college students. Cochrane Database Syst Rev.

[23] Neighbors C, Larimer ME, Lewis MA (2004). Targeting misperceptions of descriptive drinking norms: efficacy of a computer-delivered personalized normative feedback intervention. J Consult Clin Psychol.

[24] Bewick BM, West R, Gill J, O'May F, Mulhern B, Barkham M, Hill AJ (2010). Providing web-based feedback and social norms information to reduce student alcohol intake: a multisite investigation. J Med Internet Res.

[25] Khadjesari Z, Murray E, Hewitt C, Hartley S, Godfrey C (2011). Can stand-alone computer-based interventions reduce alcohol consumption? A systematic review. Addiction.

[26] Neighbors C, Lewis MA, Atkins DC, Jensen MM, Walter T, Fossos N, Lee CM, Larimer ME (2010). Efficacy of web-based personalized normative feedback: a two-year randomized controlled trial. J Consult Clin Psychol.

[27] Doumas DM, McKinley LL, Book P (2009). Evaluation of two Web-based alcohol interventions for mandated college students. J Subst Abuse Treat.

[28] Rooke S, Thorsteinsson E, Karpin A, Copeland J, Allsop D (2010). Computer-delivered interventions for alcohol and tobacco use: a meta-analysis. Addiction.

[29] Bewick BM, Trusler K, Barkham M, Hill AJ, Cahill J, Mulhern B (2008). The effectiveness of web-based interventions designed to decrease alcohol consumption--a systematic review. Prev Med.

[30] Babor TF, Higgins-Biddle JC, Saunders JB, Monteiro MG (2001). World Health Organization.

[31] McCambridge J, Thomas BA (2009). Short forms of the AUDIT in a Web-based study of young drinkers. Drug Alcohol Rev.

[32] Turner C (1990). How much alcohol is in a 'standard drink'? An analysis of 125 studies. Br J Addict.

[33] Funk M, Wutzke S, Kaner E, Anderson P, Pas L, McCormick R, Gual A, Barfod S, Saunders J, World Health Organization Brief Intervention Study Group (2005). A multicountry controlled trial of strategies to promote dissemination and implementation of brief alcohol intervention in primary health care: findings of a World Health Organization collaborative study. J Stud Alcohol.

[34] Roche AM, Stubbs JM, Sanson-Fisher RW, Saunders JB (1997). A controlled trial of educational strategies to teach medical students brief intervention skills for alcohol problems. Prev Med.

[35] Pope C, van Royen P, Baker R (2002). Qualitative methods in research on healthcare quality. Qual Saf Health Care.

[36] Kitzinger J (1995). Qualitative research. Introducing focus groups. BMJ.

[37] Mortelmans D (2007). Handboek Kwalitatieve Onderzoeksmethoden.

[38] King JA (1998). Focus Group Kit, volumes 1-6.

[39] Van Hal G, Rosiers J, Bernaert I, Hoeck S (2007). Hogere Sferen?, volume 1. Een Onderzoek naar het Middelengebruik bij Antwerpse Studenten.

[40] Rosiers J, Hublet A, Van Damme J, Maes L, Van Hal G (2011). Hogere Sferen?, volume 2. Een Onderzoek naar het Middelengebruik bij Vlaamse Studenten.

[41] Perkins HW (2002). Social norms and the prevention of alcohol misuse in collegiate contexts. J Stud Alcohol Suppl.

[42] Neighbors C, Dillard AJ, Lewis MA, Bergstrom RL, Neil TA (2006). Normative misperceptions and temporal precedence of perceived norms and drinking. J Stud Alcohol.

[43] Perkins HW, Craig DW (2006). A successful social norms campaign to reduce alcohol misuse among college student-athletes. J Stud Alcohol.

[44] Dams-O'Connor K, Martin JL, Martens MP (2007). Social norms and alcohol consumption among intercollegiate athletes: the role of athlete and nonathlete reference groups. Addict Behav.

[45] Lewis MA, Neighbors C (2004). Gender-specific misperceptions of college student drinking norms. Psychol Addict Behav.

[46] DeJong W, Schneider SK, Towvim LG, Murphy MJ, Doerr EE, Simonsen NR, Mason KE, Scribner RA (2006). A multisite randomized trial of social norms marketing campaigns to reduce college student drinking. J Stud Alcohol.

[47] DeJong W, Schneider SK, Towvim LG, Murphy MJ, Doerr EE, Simonsen NR, Mason KE, Scribner RA (2009). A multisite randomized trial of social norms marketing campaigns to reduce college student drinking: a replication failure. Subst Abus.

[48] Turner J, Perkins HW, Bauerle J (2008). Declining negative consequences related to alcohol misuse among students exposed to a social norms marketing intervention on a college campus. J Am Coll Health.

