# Supplementary material for: How is an Electronic Screening and Brief Intervention Tool on Alcohol Use Received in a Student Population? A Qualitative and Quantitative Evaluation
Source: J Med Internet Res. 2012 Apr 23;14(2):e56. doi: 10.2196/jmir.1869 (PMC3376508; doi:10.2196/jmir.1869)
Supplement: Supplementary file 1 [file jmir_v14i2e56_app1.pdf]

Appendix 1: Script for focus group discussions

Questions*	Remarks/ rationale
Introduction: "Hello everybody, my name is X and this is Y. I will conduct the discussion and Y will observe and take notes. We invited you all to discuss the website www.eentjeteveel.be**. I will ask you several open questions. Your personal opinions and view are very important for us. There are no right or wrong answers. Please feel welcome to express yourself freely during the discussion.<br>This conversation will be recorded on tape. This is only for purpose of the research, only Y and I will listen to the tape. No names or personal information will be used in the report.<br><br>Some practical issues: the discussion will last for about one hour. We ask you to please switch off your mobile phones. Please give everyone the chance to express their opinion during the conversation. You can address each other when expressing your opinion, we are only here to assist in the discussion. Is everything clear about the course of the focus group discussion?	Before starting the focus group discussion, all participants were informed about the purpose of the discussion, confidentiality and practical issues
IQ: Please share your name and your study subject?	For acquaintance with the participants and to break the ice
IQ: How did you get to know the website www.eentjeteveel.be?	For further acquaintance with the participants and gradually focusing the discussion on the study topic
SQ: Which parts of the website did you visit?<br>SQ: How much time did you spend on the website?<br>SQ: Did you check out the information menu/the test/other parts?	The sub-questions to the introduction question aimed to assess the amount of time the participants had spent on the website and how profoundly they had explored the different parts of the website
TQ: How did you experience your visit to the website?	Experiences, motivation and interest of the participants in the website were explored
SQ: Did you find your visit to the website to be meaningful?<br>SQ: How motivated were you to visit the website (and its different parts)?<br>SQ: What motivated you to visit the website (and its different parts)?	Only when the discussion had stagnated were these sub questions used
TQ: What was your general impression of the website?	To evaluate positive and negative aspects of the website
SQ: What are the positive and negative aspects of the website according to you?<br>SQ: Are the texts presented on the website clear to you?<br>SQ: What's your opinion on the interface of the website?<br>SQ: What's your opinion on the information that is provided on the website? Is it useful? Why (not)?<br>SQ: After your visit, did you feel like discussing the content of the website with others?	These sub-questions were used to further explore the positive and negative impressions of the website as experienced by the participants.
SQ: Would you use the website to address alcohol issues with your friends? Why (not)? How?<br>SQ: Could the website be used by parents to address alcohol issues with their children? Why (not)? How?	This subject was mentioned spontaneously in the first focus group discussion as an important subject and was therefore added to the script for all following focus group discussions. It explores the possible use of the website for other target groups than initially planned (i.e. students).
KQ: How did the website help you to think about your alcohol use?<br>KQ: How did the website help you to change your alcohol use?	To explore in an open-ended question how the website could help the participants think about their alcohol use, as a first step in behavioral change. Also, the intention to change their behavior was explored.
SQ: What should be changed in the website to make you think about your alcohol use?<br>SQ: Did the website motivate you to search for more information on the subject?<br>SQ: Has the website motivated you to pay more attention to your own alcohol use?<br>SQ: Has the website motivated you to pay more attention to the alcohol use of others?<br>SQ: What do you believe is the role of the internet in finding information on this subject?<br>SQ: If you did not feel addressed by the website, who should be, according to you?<br>SQ: How could the looks of the website have influenced its potential impact?<br>SQ: Would it make a difference if the website looked different/better?	These sub-questions were used to further explore the possible impact of the intervention on the participants and their behavior.<br>The role of internet as a medium to provide information on this subject and to offer help was added as a subject for discussion.<br>A question was added to stimulate the participants to think hypothetically about who should be addressed by the website (apart from themselves), because this theme had emerged from the first discussion.<br>The last two questions were added to the script after this was spontaneously mentioned in the first two focus group discussions. These explored the possible influence of the appearance of the website on its impact.
CQ: Are there any other subjects you would like to discuss concerning the website?	At the end of the focus group discussion, the participants were given the opportunity to add remarks or suggestions

*IQ: introduction question, SQ: sub-questions, TQ: transition question, KQ: key question, CQ: closing question
** Dutch, translation in English (approximately): one-too-many
